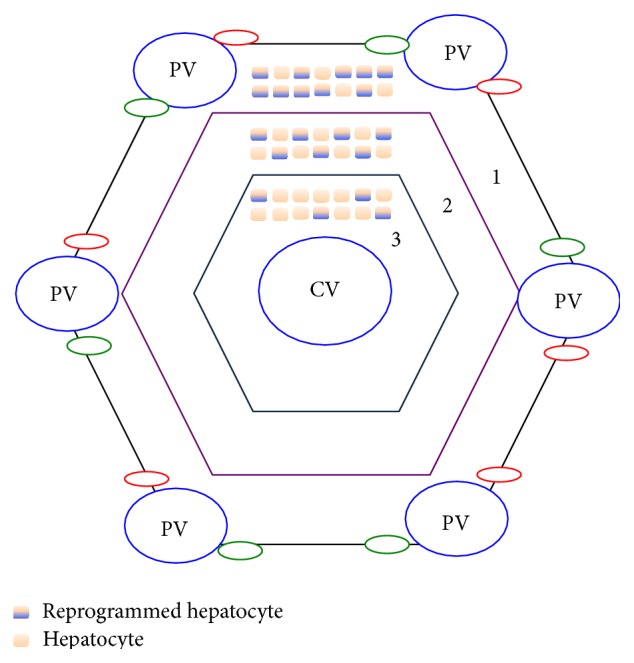# Advances in Liver Regeneration: Revisiting Hepatic Stem/Progenitor Cells and Their Origin

**DOI:** 10.1155/2016/7920897

**Published:** 2015-12-20

**Authors:** Ali-Reza Sadri, Marc G. Jeschke, Saeid Amini-Nik

**Affiliations:** ^1^Institute of Medical Science, University of Toronto, Toronto, ON, Canada M5S 1A8; ^2^Sunnybrook Research Institute, Toronto, ON, Canada M4N 3M5; ^3^Ross Tilley Burn Centre, Sunnybrook Health Sciences Centre Division of Plastic Surgery, Department of Surgery, University of Toronto, Toronto, ON, Canada M4N 3M5

## Abstract

The liver has evolved to become a highly plastic organ with extraordinary regenerative capabilities. What drives liver regeneration is still being debated. Adult liver stem/progenitor cells have been characterized and used to produce functional hepatocytes and biliary cells *in vitro*. However, *in vivo*, numerous studies have questioned whether hepatic progenitor cells have a significant role in liver regeneration. Mature hepatocytes have recently been shown to be more plastic than previously believed and give rise to new hepatocytes after acute and chronic injury. In this review, we discuss current knowledge in the field of liver regeneration and the importance of the serotonin pathway as a clinical target for patients with liver dysfunction.

## 1. Introduction

In the United States, chronic liver disease and cirrhosis are 12th in cause of death, claiming 30,000 lives annually [1]. In the 45 to 54 and 55 to 64 age cohorts, chronic liver disease and cirrhosis are listed as the 4th and 7th leading cause of death, respectively. Thus, liver disease and cirrhosis remain a prominent disorder without many treatment options. Considering the liver's diverse range of essential functions [2] and magnificent capacity to regenerate mostly in acute settings, it is imperative that we gain a deeper understanding of liver regeneration after acute and chronic injury in order to target pathways for therapeutic benefits.

The liver's response to injury is puzzling as it has multiple methods of regeneration depending on the type of injury. A 2/3 partial hepatectomy (PHx) promotes regeneration of the liver through hepatocyte hypertrophy and proliferation [3]. However, when the liver is subjected to toxins, there is an accumulation of hepatic ductal cells also known as “oval cells,” which restore liver function by replacing liver parenchymal cells. We will discuss the current hypotheses that have been proposed for liver regeneration and will highlight the role of different cell types during repair and regeneration.

## 2. Liver Anatomy

**Figure 1:**
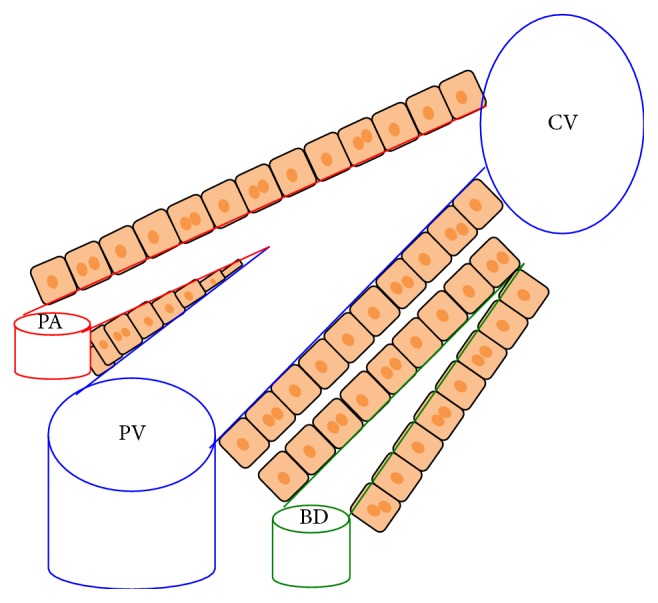


**Figure 2:**
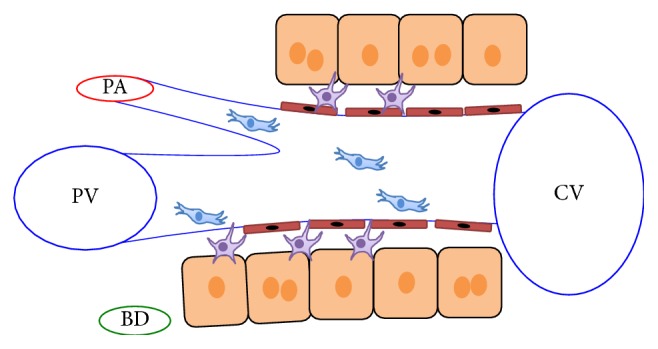


The adult liver is composed of lobes that contain parenchymal and nonparenchymal cells. Parenchymal cells include hepatocytes and cholangiocytes while nonparenchymal cells consist of Kupffer cells, stellate cells, and endothelial cells. The lobes are further dissected into lobules, which are the functional units of the liver. The lobules are polygonal in shape with portal venules, arterioles, and bile ducts at the borders and a central vein in the center (Figure 1). Hepatocytes are known for their metabolic properties and ability to detoxify blood. They are also known as the main cell type of the liver, encompassing 80% of the mass. The cholangiocytes line the bile ducts and form the biliary tree. They act as a barrier to prevent bile from damaging the rest of the liver. Hepatic stellate cells (HSCs) are the resident mesenchymal stem cells in the liver and reside in the space of Dissé. In their quiescent state, they store vitamin A, but upon injury they differentiate into myofibroblasts. In contrast, Kupffer cells are the resident macrophages located in the sinusoidal lumen which work to detoxify blood and release various cytokines (Figure 2).

## 3. Liver Development 

Understanding the process of liver development is important when studying liver regeneration, since effective regeneration involves recruitment of similar developmental pathways. The endoderm gives rise to the main cell types of the liver, hepatocytes and cholangiocytes. During gastrulation the endoderm germ layer forms a primitive gut tube that is divided into foregut, midgut, and hind gut. The foregut gives rise to the hepatic diverticulum, which will eventually give rise to the liver and gall bladder. Specification of the liver involves signalling from the surrounding cardiac mesoderm, septum transversum, and endothelium [4–6]. The cardiac mesoderm promotes FGF signalling while the septum transversum mesenchyme (STM) promotes BMP signalling, which collectively induces liver specification [6]. Hepatic fate is determined once liver genes such as albumin are expressed in hepatoblasts. Once hepatic specification is complete, the hepatic epithelium thickens and eventually breaks through its basement membrane to invade the STM, upon which hepatoblasts proliferate and enter the STM to form the liver bud [7]. Hematopoeitic cells proceed to invade the liver bud, making hematopoiesis the main function of the fetal liver, as it quickly morphs into a mature liver.

## 4. Important Signalling Pathways in Liver Development

Development of the liver requires coordination between several signalling pathways, such as transforming growth factor *β* (TGF-*β*), Wnt, fibroblast growth factor (FGF), Notch, and bone morphogenetic protein (BMP) [8, 9]. Further discussion of these pathways is outside the scope of this review however; they are also active during regeneration in the adult organ [10].

Certain mechanisms such as development and regeneration of the adult liver are still unclear. Although the liver is recognized as a highly regenerable organ, the activity of what we consider “stem cells” is very low during homeostasis and after an acute injury, supported by numerous studies [11–13]. Whether liver progenitor cells are not involved during acute regeneration or we are lacking markers that clearly specify the progenitor cells is not clear. Using available markers, it seems that stem/progenitor activity is not routinely observed in the liver until chronic injury has occurred. Chronic injury to the liver typically impairs hepatocyte proliferation, resulting in hepatic progenitor cells becoming activated, which give rise to the epithelial cells of the liver, such as hepatocytes and cholangiocytes.

### 4.1. Nomenclature of Liver Stem/Progenitor Cells

Liver stem/progenitor cells have various titles such as “ductular hepatocytes,” “intermediate hepatobiliary cells,” “atypical ductular proliferation,” or often times “oval cells.” Oval cells were identified in rodent studies, which describe them as small in size, oval-shaped nucleus, limited cytoplasm, and a lack of basement membranes [14, 15]. They appear in models of toxin-induced injury and hepatocarcinogenesis [16, 17]. Where these cells are derived from is still a topic of debate but, using markers such as* Sox9*,* Foxl1* Epcam, PanCK, and MIC1-1C3, the consensus is that they emerge from BECs in the Canals of Hering [18].

### 4.2. Tracing the Fate of Stem/Progenitor Cells

**Figure 3:**
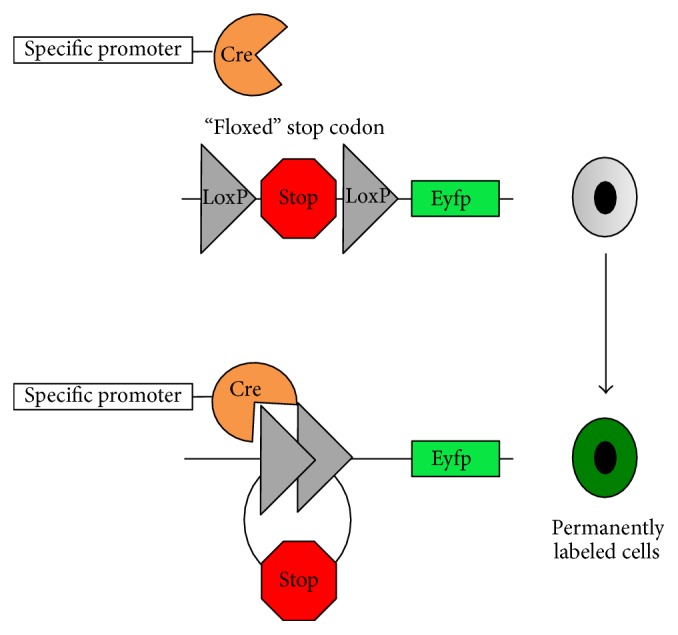


The study of HPCs involves lineage tracing tools to identify the location and fate of these cells upon injury. A frequent method used to trace cells is through an inducible cre-loxp system, which uses a cell specific promoter along with a reporter gene such as GFP or LacZ to track the population of interest (Figure 3). Developing these mice involves crossing a mouse with an inducible Cre-recombinase enzyme that is a cell specific promoter with a mouse that has a reporter gene downstream of the floxed stop codon. Cre-recombinase targets that stop codon, which is excised allowing the progeny of the two mice to permanently express the reporter gene specifically in the cell population that expresses the promoter. This method allows for tracking of all the progeny through multiple cycles as the lineage is tagged with a marker.

### 4.3. Liver Responses after Acute Injury

**Figure 4:**
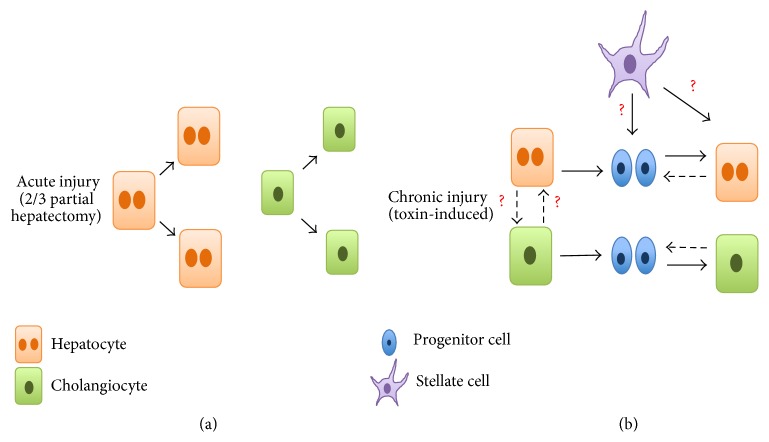


After a 2/3 PHx, the regenerative process involves proliferation and hypertrophy of existing hepatocytes rather than any significant contribution from stem/progenitor cells (Figure 4(a)). This robust regenerative capacity of hepatocytes suggests that stem/progenitor cells may not be required for liver regeneration. However, although PHx is a useful model, it does not fully encompass what is observed in the clinic. This model does not involve significant hepatocyte death and subsequent inflammation and fibrosis, which are observed in many human liver diseases [19]. Lacking the apoptotic cascades and inflammatory responses is a hallmark of 2/3 PHx and therefore may explain why stem/progenitor cells do not have a role in this compensatory response.

### 4.4. Liver Regeneration after Toxin-Induced Injury

Some studies suggest that there is an alternative mechanism to hepatocyte regeneration, which involves facultative stem cells (FSCs). FSCs are differentiated cells that attain a “stem cell-like” state after certain types of injury (Figure 4(b)). Initially unipotent during homeostatic conditions, FSCs adopt multipotential characteristics when homeostasis is disrupted. This hypothesis came from rat studies in which the liver had to regenerate after exposure to several hepatotoxic carcinogens. It is believed that these cells appear when hepatocyte proliferation is impaired. In mice, these cells are still observed even with hepatocyte proliferation. These facultative stem cells are oval-shaped with biliary properties [20]. Nevertheless, this adds evidence, highlighting the plasticity of liver cells.

### 4.5. Liver Responses after Chronic Injury

During chronic liver injury, there is a substantial increase in hepatic stem/progenitor cells, which are believed to be derived from within the portal field and expand into the parenchyma [21]. In humans with chronic liver disease, the expansion of biliary-like cells or oval cells is associated with severity of the disease [22]. In rodent models, these oval cells have the capacity to differentiate into hepatocytes and cholangiocytes [23]. Thus, progenitor cells maybe an important population to target to improve liver function in chronically diseased livers. Despite this, the existence and nature of hepatic stem cells are still questioned. Furthermore, there is no specific marker or identifiable stem cell niche in the liver. In fact, studies show that mature cells in the liver can contribute to regeneration after a partial hepatectomy without any stem or progenitor cell activity. Hepatocytes give rise to new hepatocytes through hypertrophy and proliferation, as well as producing paracrine signals that stimulate proliferation of other cell types [3]. Once hepatocyte proliferation is impaired such as in the 2-AAF/PHx model done in rats, there is a large accumulation of oval cells that express biliary markers but also hepatocyte markers such as albumin and *α*-fetoprotein [24]. Expression of transcription factors in hepatoblasts during development has also been observed in oval cells upon chronic injury, suggesting similarities in function between the two cells [24].

### 4.6. The Role of* Sox9* Positive Cells after Liver Injury

Sex determining region Y (*Sox9*) is a marker used to identify progenitor cells and biliary cells in the liver. Furuyama et al. (2011) developed* Sox9*-GFP and* Sox9*-LacZ mice, in which all* Sox-9*+ cells and their progeny will express either GFP or LacZ [25]. They showed that when healthy animals were left alone for 12 months, the epithelial cells were replaced by* Sox-9*+ cells due to the increase in cells expressing GFP or LacZ. This directly challenges what is observed in other studies that suggest liver homeostasis is maintained through division of mature epithelial cells [12]. Furthermore, they show that* Sox9*+ cells are the source of epithelial cells after chronic injury due to repeated CCl_4_ injections or bile duct ligation (BDL). A limitation in this study is that the use of tamoxifen has been shown to induce expression of ductal markers, such as* Sox9* in mature hepatocytes and mature hepatocytes themselves are able to express transcription factors associated with biliary cells [11, 26]. Having right controls can minimize these limitations.

Considering the aforementioned limitations, through clonal tracing of* Sox9*+ cells and optimized administration of tamoxifen to avoid ectopic* Sox9* expression, Tarlow et al. show that* Sox9*+ cells do not contribute to liver homeostasis under normal conditions or to hepatocyte replacement in traditional oval cell mediated injury models [27]. Clonal relationship between* Sox9*+ cells and the two epithelial liver lineages was established. Using* Sox9*+ cells with a multicolour fluorescent confetti reporter, Tarlow et al. showed that <1% of* Sox9*+ cells contributed to the hepatocyte regeneration in injury models such as DDC, CDE (Choline-Deficient Ethionine), and repeated carbon tetra-chloride (CCl_4_) injections.

Cell lineage studies using* Sox9* and* Foxl1* reporters show that stem/progenitor cells exist but this remains a highly controversial topic [25, 28].

### 4.7. The Role of* Lgr5* Positive Cells after Liver Injury

**Figure 5:**
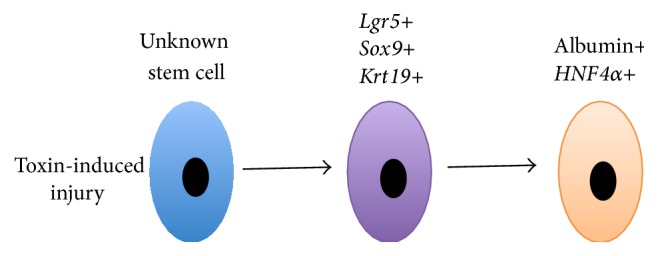


*Lgr5*, a Wnt target gene, is expressed in proliferating stem cells. Lineage tracing studies show that, in the damaged liver,* Lgr5*+ cells can generate hepatocytes and bile ducts* in vivo* [29]. Isolating* Lgr5*+ cells and expanding them to form functional hepatocytes saves Fah^−/−^ mice [29]. Although these findings are very promising, the authors do not address the source of* Lgr5*+ cells, as they are not present in the normal adult liver. It may be possible that upon injury and during the process of differentiation,* Lgr5*− progenitor cells attain expression of* Lgr5* and show there is a subset of hepatocytes that are able to go back into a progenitor state. An alternative explanation could be that the true resident liver stem cell is yet to be defined as* Lgr5*+ cells are likely higher up in the lineage (Figure 5).

Thus, the source of new epithelial cells and the underlying mechanisms in liver regeneration are more complex than previously thought. An alternative explanation to how hepatocytes regenerate after chronic injury is required as findings with* Sox9*+ and* Foxl1*+ positive cells are conflicting and* Lgr5*+ cells are only part of the liver stem cell conundrum.

### 4.8. The Role of Mature Hepatocytes after Liver Injury

To understand regeneration and organ repair, we need to look beyond just humans and rodent models. Amphibians have shown a remarkable capacity to regenerate multiple organs such as limbs, spinal cord, retina, and some sections of the heart and brain [30]. A key source of cells during their regenerative response is through dedifferentiation of cells and forming a progenitor cell pool. In mammals, replacement of cells is typically done through proliferation or differentiation of stem or progenitor cells. Reprogramming of cells* in vivo* has been done but through exogenous factors [31].

Through the use of modern lineage tracing tools, recent findings suggest that mature hepatocytes exhibit greater plasticity than previously known, as they are able to transition into a progenitor-like state to give rise to new hepatocytes after chronic injury [27, 32]. These progenitor-like cells are derived from mature hepatocytes and exhibit properties similar to oval cells. It was previously believed that hepatocytes are terminally differentiated and only proliferate after acute injury, such as a partial hepatectomy. Several groups have revealed hepatocytes have the capacity to “transdifferentiate” into ductal biliary epithelial cells after injury [33–36].

Cellular plasticity is associated with Epithelial-to-mesenchymal transition (EMT) that is observed in the transition of mature hepatocytes into biliary-like progenitors. This transition is shown through expression of mesenchymal markers such as* Vim* [37] and* Zeb1* [38] in addition to stem/progenitor markers* Sox9*,* c-kit*,* Fn14*, and* Cd44* [27]. Numerous pathways have been shown to promote conversion of hepatocytes to progenitor cells such as Wnt/*β*-catenin, Tgf-*β* [38], Notch [39], and hedgehog signalling [40]. Gene analysis studies also point to Hippo/Yap pathway as another key regulator of hepatocyte dedifferentiation and maintenance of mature state [41].

Hippo/YAP signaling appears to play an essential role in determining cellular fate in the mammalian liver [41]. Increasing YAP activity in mature hepatocytes promotes dedifferentiation of these cells into a progenitor state. Notch signaling is an important downstream target of YAP in liver cells [42]. Through lineage tracing of hepatocytes, studies have shown that,* in vivo*, approximately 75% of mature hepatocytes have the ability to change their fate after YAP activation [41]. This suggests that most hepatocytes have an intrinsic ability to dedifferentiate into progenitor cells and give rise to new epithelial cells in the liver.

Furthermore, during ontogeny, Notch has a vital role in determining the fate of hepatoblasts to cholangiocytes [43]. This appears to be recapitulated in the adult liver to promote hepatocyte-to-BEC reprogramming. Reprogramming of cells may be a standard response to biliary cell injuries such as DDC treatment or BDL [35]. The role of Notch signalling and hepatocyte plasticity goes beyond regeneration as it is also observed in liver cancer.

It was originally thought that the source of intrahepatic cholangiocarcinomas is biliary epithelial cells [44]. However, hepatocytes have been shown to be a significant source of cholangiocarcinoma due to the observation that masses are formed where mainly hepatocytes reside [45]. According to lineage studies, there appears to be transdifferentiation of hepatocytes into biliary cells through upregulation of Notch and AKT signalling in a model of cholangiocarcinoma [46].

Transdifferentiation of hepatocytes into BECs has also been suggested in humans [47]. In diseases such as primary biliary cirrhosis and chronic biliary obstruction, there is upregulation of* HNF3β* in hepatocytes, which is normally expressed in BEC under healthy conditions. Furthermore, hepatocyte associated transcription factors such as* HNF4α* and* HNF6* appeared in BEC after massive hepatic necrosis and chronic hepatitis C virus infection [47]. The data observed in human studies supports findings in rodent models, which show higher expression of hepatocyte associated transcription factors in biliary cells [47, 48]. Oval cells initiate expression of transcription factor* HNF4α* and eventually increase in size to become “small hepatocytes” and ultimately become mature hepatocytes.

It appears that hepatocytes have the capacity to function as facultative stem cells and rescue the BECs in response to injury, which causes impaired BEC proliferation [33]. Similar observations are reported when hepatocyte proliferation is impaired, such as in the 2-AAF/PHx model done in rats where there is increased proliferation of biliary derived progenitor cells which differentiate into hepatocytes [49]. These studies suggest that hepatocytes and BECs may act unselfishly and save the other cell population when one cannot save itself.

Overall, with regard to stem cell biology, it is clear that the liver is unlike any other organ. In contrast to the skin or intestine, the studies mentioned above suggest that the liver does not have tissue specific stem cells, which maintain homeostasis and regenerate the organ upon injury. Instead, the liver has mature epithelial cells that are facultative stem cells, which lay dormant until there is toxin-mediated injury. This results in the subsequent transdifferentiation of either BECs or hepatocytes into the required epithelial cell type. Moreover, dedifferentiation is another suggested mechanism, which is observed in hepatocytes that become oval-like cells and differentiates back into hepatocytes [27, 35]. Whether these hepatocyte derived oval-like cells give rise to BECs is still unknown.

## 5. Hepatocyte Heterogeneity

Although the heterogeneity of hepatocytes is well documented in terms of metabolism [50], it is still not understood if there is a subpopulation of hepatocytes that exhibit higher plasticity than others. Hepatocytes that are located adjacent to the portal venules are in “Zone 1.” It is believed that “Zone 1” and to a lesser extent “Zone 2” hepatocytes have a higher efficiency in undergoing cellular reprogramming compared to hepatocytes around central veins also known as “Zone 3” [26, 35, 51] (Figure 6). It is interesting to note that the Notch pathway gets activated in all the zones encompassing greater than 95% of hepatocytes, but Zone 1 and Zone 2 hepatocytes undergo ductular reactions. This suggests that there are either missing signalling pathways in Zones 1 and 2 or inhibitory pathways in Zone 3 that prevent this reprogramming response. Furthermore, the extracellular milieu may also vary between the zones as hepatocytes in each zone have different metabolic functions [52].

### 5.1. The Role of Myeloid Lineage Cells after Liver Injury: Drivers of Regeneration?

Myeloid cells, in particular macrophages, have recently become a hot topic in regeneration. First, they were shown to be a major component of limb regeneration in amphibians. In fact, without macrophages, limb regeneration is blocked, as there is a lack of dedifferentiation and formation of progenitor cells [30]. Over the years, macrophages have been shown to be highly plastic and are able to change their phenotype based on environmental cues [53]. During liver fibrosis, these cells exist in a spectrum of states depending on which phase of repair the injury is in. For example, macrophages have a profibrotic role during the early phase of injury in the liver, while in the late phase they become antifibrotic and secrete anti-inflammatory factors [54].

Furthermore, macrophages have been linked to secretion of Wnt ligands during liver regeneration [55, 56]. Phagocytosis of hepatocyte debris triggers wnt3a secretion from macrophages which inherently promotes hepatocyte fate in HPCs [55]. Depletion of macrophages via liposomal clodronate during hepatocyte regeneration in a chronic injury model resulted in a shift in the fate of HPCs from hepatocytes to cholangiocytes [55]. In acute injury models such as 2/3 PHx, ablation of macrophages results in impaired regeneration due to lack of key proliferative cytokines such as IL-6 and TNF [57]. Ablating cd11b+ cells followed by a PHx show that monocytes and macrophages are essential for angiogenesis and liver mass regeneration and survival [58]. Furthermore, normal regeneration kinetics of the liver after partial hepatectomy is contingent upon Wnt ligands secreted by Kupffer cells, which promote Wnt/*β*-catenin signaling in hepatocytes [56]. When the gene* Wntless* is knockdown in macrophages, there is a 33% reduction in S-phase hepatocytes and hepatocyte mitosis, which is related to the diminished *β*-catenin-TCF4 complex and Cyclin-D1 expression at 40 hours. Thus, Kupffer cells have an essential role in promoting hepatocyte proliferation in a coordinated fashion through secretion of Wnt ligands.

### 5.2. The Role of Hepatic Stellate Cells (HSCs) after Liver Injury

In their quiescent state, HSCs store retinoids. They are known as the resident mesenchymal stem cells of the liver and typically reside in between sinusoidal endothelial cells and hepatocytes in the space of Dissé [59]. Upon liver injury due to hepatic toxins or viral infection there is activation of HSCs, which causes them to differentiate into myofibroblasts [60]. Myofibroblasts are the primary producers of collagen in the liver and have a central role in liver fibrosis [60]. HSCs are known to be the main producers of TGF-*β* in the liver, which is a key factor in stopping regeneration once the appropriate liver mass is achieved. Furthermore, HSCs have already been shown to influence the differentiation of progenitor cells into bile duct cells [55]. Whether HSCs contribute to the progenitor cell population or they are mainly a niche provider is still in debate.

HSCs are under study to determine if they are involved in stem/progenitor cell based liver regeneration.* In vitro*, activated stellate cells can develop into hepatocyte-like cells [61, 62].* In vivo* experiments using cell lineage tracking with* Gfap* and* Acta2* as promoters show that activated stellate cells contribute to the hepatic progenitor pool [40, 63, 64]. Using lineage tracing to track transplanted HSCs in damaged rat livers, Kordes et al. show that HSCs can graft to the injured liver and contribute to tissue regeneration by developing into progenitor-like cells and epithelial cells [65]. Others suggest that HSCs give rise to myofibroblasts only and not to liver epithelial cells [60, 66].

### 5.3. The Role of Liver Sinusoidal Endothelial Cells (LSECs) after Liver Injury

Another cell type that has recently attracted attention is LSECs. This population is critical for activating proregenerative or profibrotic responses in the liver through angiocrine factors [67]. The effect LSECs has on liver regeneration is context dependent. For example, after an acute injury such as a 2/3 PHx, there is upregulation of CXCR7 along with CXCR4, which have a combined effect of promoting upregulation of transcription factor inhibitor of DNA binding 1 (Id1) [67, 68]. This cascade of events promotes production of Wnt2 and HGF, which are proregenerative angiocrine factors, and triggers regeneration. After chronic injury, such as by repeated injections of CCl_4_ or BDL, FGFR1 signalling promoted greater CXCR4 signalling over CXCR7, which in turn stimulates proliferation of desmin+ stellate cells. Consequently, a profibrotic phenotype is observed [67].

The temporal response of regeneration after a PHx is regulated through angiopoietin 2 (Ang2) secretion by LSECs [69]. Production of Ang2 is downregulated during the early phase of regeneration (days 1–3), which is associated with decreased expression of TGF-B, an antiproliferative factor, and increased expression of cyclin D1, thus promoting hepatocyte proliferation. In the later phase of regeneration (days 4–7), Ang2 levels rise, which promotes higher expression of VEGFR2 and Wnt2, enabling proliferation of LSECs [69, 70]. Understanding what pathways promote fibrosis over regeneration will allow us to develop therapeutics to counter these factors.

## 6. Novel Pathways: Serotonin

Serotonin (5-HT) is known predominantly for its role as a neurotransmitter and is also a hormone with essential extraneuronal functions [71, 72]. Serotonin is transported to various sites of injury and inflammation via platelets. The role of platelet-derived serotonin was shown in an experiment using GPIb*α*, a platelet specific antibody [73]. Mice treated with this drug and then subjected to a PHx showed a significant reduction in hepatocyte proliferation [73]. Furthermore, antagonizing 5-HT_2A_ and 5-HT_2B_ receptors impaired liver regeneration. Mice that lack the rate-limiting enzyme for serotonin synthesis, tryptophan hydroxylase 1, also had impaired regeneration [74]. Treating mice with serotonin-loaded platelets saved these mice [73]. Interestingly, numerous clinical studies showed that poor circulating platelet counts were linked to hindered liver regeneration after liver resection [75, 76]. In combination with the multiple animal studies done, 5-HT definitely has a role in liver regeneration [73, 77–80]. Recently, intraplatelet levels of 5-HT have been shown to correlate with liver regeneration in humans [81]. This suggests that levels of intraplatelet 5-HT prior to liver resection maybe a clinical predictor of postoperative liver dysfunction. However, the type of injury needs to be taken into consideration. In chronic injury mouse models such as BDL, blocking the 5-HT_2B_ receptor on HSCs actually reduced fibrosis and enhanced liver function [82]. As discussed above, HSCs are essential for stopping regeneration through TGF-B secretion, which is mediated through the 5-HT_2B_ receptor. Thus, blocking this receptor when fibrosis overrides regeneration maybe beneficial for patients suffering from liver fibrosis and cirrhosis. Pharmaceutical drugs targeting the serotonin pathway and specifically the 5-HT_2B_ receptor are deemed clinically safe for humans and maybe have reparative benefits for patients with liver disease.

## 7. Conclusion 

The liver's regenerative capacity has remained a puzzle for centuries. It is capable of fully regenerating itself from a wide range of injuries and toxins. This regenerative response is highly complex and involves crosstalk between numerous resident and recruited cells. There are several theories to how the liver regenerates with no consensus in sight. The discrepancies between the studies discussed maybe due to different models used, extent of damage, time of injury, and species-specific differences. However, there is consistency between rodent models [83] and humans [84] as both show that ductular reactions occur, which contributes to regeneration. However, questions remain about how instrumental each cell type is to the different phases of liver regeneration. Moreover, the idea of hepatocyte plasticity and heterogeneity is intriguing because it suggests that these cells or a subset of cells have the capacity to become progenitor cells and assist with regeneration. This hypothesis is garnering attention and further lineage studies are required to validate this idea.

Whether there are resident or recruited liver stem cells, dedifferentiation or transdifferentiation of hepatocytes and biliary cells, or contribution of HSCs to the progenitor pool is still up for debate. One thing that is certain is that the liver has evolved to become a highly plastic organ, which is able to repair itself through contribution of numerous cell types and signalling pathways.

## Figures and Tables

**Figure 6:**